# Improving the effectiveness of performance feedback by considering personality traits and task demands

**DOI:** 10.1371/journal.pone.0197810

**Published:** 2018-05-22

**Authors:** Victor Swift, Jordan B. Peterson

**Affiliations:** Department of Psychology, University of Toronto, Toronto, Ontario, Canada; University of Zurich, SWITZERLAND

## Abstract

Although performance feedback is widely employed as a means to improve motivation, the efficacy and reliability of performance feedback is often obscured by individual differences and situational variables. The joint role of these moderating variables remains unknown. Accordingly, we investigate how the motivational impact of feedback is moderated by personality and task-difficulty. Utilizing three samples (total *N* = 916), we explore how Big Five personality traits moderate the motivational impact of false positive and negative feedback on playful, neutral, and frustrating puzzle tasks, respectively. Conscientious and Neurotic individuals together appear particularly sensitive to task difficulty, becoming significantly more motivated by negative feedback on playful tasks and demotivated by negative feedback on frustrating tasks. Results are discussed in terms of Goal-Setting and Self Determination Theory. Implications for industry and education are considered.

## Introduction

Performance feedback (PF) is an invaluable, yet unreliable, tool. Integral to academic and industrial domains, PF is used to guide behavior by conveying an appraisal of performance [1, 2]. Although PF employed in feedback interventions can improve motivation and performance [3–10], almost 1/3rd of studies have failed to identify PF as a significant positive force [11].

The inconsistent efficacy of PF is likely due to the influence of extraneous variables. Indeed, a recent meta-analysis suggests that the efficacy of feedback is strongly determined by two variables: task features (e.g. difficulty) and personality [1]. Accordingly, understanding the influence of these two variables is essential to the project of making PF more reliable. Although individually well understood, the joint influence of task features and personality on PF efficacy remains largely unknown. Thus, we herein explore how the motivational impact of PF is moderated by personality and task features, together.

In terms of task features, we narrow our focus to task difficulty, understood as the degree to which a task is frustrating and effortful (independent of skill). Difficult tasks inherently undermine motivation whereas playful (i.e. enjoyable and easy) tasks inherently promote motivation [12, 13].

According to Self Determination Theory (SDT), threats to basic needs such as competence undermine motivation, whereas the promotion of these same needs increases intrinsic motivation [14]. Therefore, qualities that make a task difficult (such as deadlines or pressured competition), pose a considerable threat to competence and thereby undermine motivation [15]. Likewise, qualities that make a task playful (such as flexibility or exploration) promote competence [16] and thereby increase motivation.

PF differentially affects motivation on difficult vs. playful tasks. Specifically, negative feedback has been found to decrease motivation on difficult tasks [17, 18] and increase motivation on playful tasks [19, 20]. This phenomenon can be understood by combining Goal Setting Theory with SDT [21].

Goal Setting Theorists posit that negative PF signals a discrepancy between performance and standards, thereby provoking one of two broad motivational reactions: commitment or abandonment. When feelings of competence are intact, people opt to resolve the discrepancy between performance and standards by increasing effort on tasks. Otherwise, the task or standard is abandoned [22]. Thus, when feelings of competence have been depleted by a difficult task, negative PF can have a demotivating effect. Alternatively, when feelings of competence have been promoted by a playful task, negative PF can have a motivating effect.

Considering that feelings of competence are central to this explanation, individual differences that modulate feelings of competence likely contribute to the motivational outcomes of *PF x task difficulty*. This notion finds support from the sole PF article we found to consider both personality and task features. Tang and Sarsfeild-Baldwin [23] considered the motivational impact of PF on easy vs. difficult tasks for individuals who scored high vs. low on a measure of self-esteem–a trait characterized by competence-related threat sensitivity [24]. These researchers found that individuals with low self-esteem were more affected by negative feedback on difficult tasks than individuals with high self-esteem. Although these findings offer support for a joint role of personality and task difficulty in PF effects, the broad nature of this interaction remains unclear. This is because self-esteem represents a single and narrow aspect of personality that remains poorly defined [25].

We herein conduct a more comprehensive examination of the *PF x task difficulty x personality* interaction by measuring the most robust personality variables known to date: the Big Five [26]. The Big Five traits represent stable, broad, and coherent individual differences in affective, behavioral, cognitive, and motivational tendencies [27]. Of the Big Five, two traits stand out as potential moderators of the motivational effect of *PF x task difficulty*: Conscientiousness and Neuroticism. Both of these traits include a dimension of competence-related threat sensitivity, and both traits have emerged as the most frequent Big Five moderators of PF effects (see below for a review) and performance motivation in general (see meta-analysis in [28]). Accordingly, we hypothesize that Conscientiousness and Neuroticism moderate the motivational effects of *PF x task difficulty*. However, due to the paucity of directly relevant research, we maintain an exploratory approach to our analyses which acknowledges the possible influence of other Big Five traits.

Before explicating expected outcomes, we review the PF literature associated with each Big Five trait, in order of abundance. Given the scarcity of PF research directly measuring the Big Five, we include studies that have measured personality variables closely related to the Big Five.

### Conscientiousness

Conscientiousness represents a tendency for self-restraint, hard work, and organization [29]. Likely due to their focus on achievement and performance [30], Conscientious people have been shown to exhibit a sensitivity to competence-related threats. Compared to people low in Conscientiousness, highly conscientious individuals experience more stress [31], tension [32], and negative affect [33] following threats to competence. In the context of SDT, this sensitivity would suggest that conscientious people are particularly vulnerable to PF effects.

Conscientiousness has been found to produce conflicting motivational effects following both negative and positive feedback. The two studies to date that have found Conscientiousness to significantly moderate the effects of negative feedback report opposite outcomes. Using scores from a computer Naval Air Defense simulation as an outcome variable, Mudgett [34] found that highly conscientious participants performed significantly better after negative feedback than after positive feedback or no feedback. Alternatively, Cianci, Klein, and Seijts [32] found that conscientious participants who received negative feedback subsequently performed worse on text comprehension and analogy questions.

Importantly, both Mudgett [34] and Cianci et al. [32] attribute these outcomes to the moderating effect of task difficulty. Cianci et al. found that highly conscientious people felt tense during the task, regardless of feedback condition. Alternatively, Mudgett found that highly conscientious participants in the negative feedback condition felt more satisfied and competent after each trial. Taken together, these findings suggest that the task that Cianci et al. used was perceived by participants as stressful and difficult, and the task that Mudgett used was perceived as playful and easy. Indeed, the manual for the performance task used by Mudgett describes the Naval simulation as a computer *game* [35].

Feedback studies employing personality measures akin to Conscientiousness (e.g. need for achievement) suggest that positive feedback facilitates motivation for individuals who are high in Conscientiousness-like traits [36–38]. However, the effect of positive feedback on Conscientiousness-proper remains ambiguous. Of the two PF studies that found a significant association between Conscientiousness and positive feedback, one found positive feedback to increase motivation [39], while the remaining study found positive feedback to decrease motivation for conscientious people [40].

### Neuroticism

Neuroticism represents a tendency to experience negative emotional states, predominantly aggression or withdrawal [41,42]. Likely due to their vulnerability and self-consciousness [43], neurotic people have been shown to exhibit a sensitivity to threats in general [44], including competence-related threats. Compared to people low in Neuroticism, highly neurotic people experience more negative affect [45, 46], and maladaptive coping [47] following threats to competence. In the context of SDT, this sensitivity would suggest that neurotic people are particularly vulnerable to PF effects.

The research based on Neuroticism-like traits, and later research measuring Big Five Neuroticism, converges to suggest that neurotic individuals are particularly responsive to negative feedback, but not positive feedback. However, the *motivational effects* of the negative feedback appear to be inconsistent for neurotic people.

Negative feedback has been found to impair motivation of people who score high on Neuroticism [47, 48], and Neuroticism-like traits, such as low self-esteem, and sensitivity to criticism [49, 50]. However, negative feedback has also been found to improve motivation for individuals who are high scorers on these same traits [37, 51, 52].

As in the case of Conscientiousness, these conflicting results may reflect differences in task difficulty. Indeed, in studies where negative feedback demotivated neurotic participants, stressful and difficult tasks were used. These tasks rushed and startled participants [48], were hard or impossible to improve on [47, 50] or represented a real-world risk [49]. In contrast, studies that found negative feedback to motivate neurotic participants used easy and enjoyable tasks–for instance, open-ended creative thinking tasks [37, 51], and simple recall tasks [52].

### Extraversion

Extraversion represents a tendency to energetically approach the social and material world–for instance, sociably, assertively, or enthusiastically [42]. Extraversion has been found to play an occasional role in feedback effects insofar as this trait is closely associated with positive emotionality. Extraverts feel better after positive feedback [53], which can result in improved performance [34]. However, extraverts report being less concerned with the polarity of feedback [54], which may explain the insignificance of this trait in most feedback research.

### Openness

The trait of Openness represents a proclivity for variation in experiences, thoughts, and feelings–for instance, through involvement in art, education or adventure [42]. Although open people are eager to utilize feedback in organizational settings [55] and report feeling that feedback is valuable [54], this trait has not emerged as a significant moderator of positive or negative feedback in any of the studies reviewed herein.

### Agreeableness

The trait of Agreeableness represents a communal and prosocial orientation, encompassed by tendencies towards politeness and compassion [41, 42]. We were unable to find any published research that found Agreeableness to be a significant moderator of feedback on performance or motivation. However, individuals with a high concern for others–a facet of Agreeableness [43]–are more likely to take initiative when they are provided with positive feedback [56].

### Hypotheses

The reviewed literature suggests that Conscientiousness and Neuroticism alone will moderate the motivational effects of *PF x task difficulty*. This is in part suggested by the relative abundance of PF literature involving these traits. This is also suggested by the implicit role of task difficulty revealed in our review of contradictory PF effects (e.g. the a-posteriori finding that negative feedback decreased motivation for conscientious and neurotic people working on apparently difficult tasks).

Because no research has directly considered the moderating role of the Big Five considered herein, an exploratory approach is favored. Nevertheless, the expected role of Conscientiousness and Neuroticism also has a theoretical basis. According to Goal Setting Theory, negative PF signals that more effort is needed to reach a standard. This signal provokes a decision-making process that results in task commitment or task abandonment [21]. Feelings of competence are a central variable in this decision-making process [14], with increased feelings of competence promoting task commitment, and decreased feelings of competence promoting task abandonment [22].

Difficult tasks undermine feelings of competence; thus, negative feedback on these tasks has a demotivating effect. Alternatively, playful tasks foster feelings of competence; thus, negative feedback on such tasks has a motivating effect. These effects are expected to be pronounced for people who are inherently sensitive to competence-related threats (i.e. conscientious and neurotic people).

Using three samples, we investigate how task difficulty and Big Five personality traits jointly moderate the motivational effects of PF. All three samples completed variations of discovery-based puzzle tasks, which are common in feedback research (e.g. [57–59]). Moreover, the feedback procedure across the three samples remains identical. Task difficulty is the only variable to differ across samples. Specifically, the puzzle task in Sample 1 was designed to be difficult, in Sample 2 to be playful, and in Sample 3 to be neutral (i.e. neither difficult nor playful).

We broadly hypothesize that personality traits will interact with feedback valence on difficult and playful tasks, but will not interact with feedback on neutral tasks. Moreover, we expect that Conscientiousness and Neuroticism will moderate the motivational effects associated with *feedback valence x task difficulty*; however, we remain open to the possibility that other personality traits may also moderate this interaction.

## Methods

### Procedure

All experiments reported herein were approved by the Research Ethics Board of the University of Toronto, and all participants provided written informed consent. Apart from the particular discovery-based puzzle tasks that were used, the procedures for Samples 1, 2 and 3 were identical. Participants were recruited through Amazon's Mechanical Turk, an online sampling platform representative of the U.S. population [60] that has repeatedly been shown to produce reliable and valid data [61, 62]. The study itself was compiled on and administered through the Qualtrics online platform. The survey began once participants had read and signed the study consent form. This form vaguely explained that the survey included a puzzle portion and a questionnaire portion, however, participants were not informed that they would receive feedback.

Upon consent, a demographics questionnaire was administered which surveyed age, gender, occupational status, educational status, income, and ethnicity. This questionnaire was followed by the precursor puzzle module and then the Big Five questionnaire. Afterwards, participants were randomly assigned to a positive or negative feedback condition and shown a corresponding non-specific false feedback prompt seemingly based on precursor puzzle performance. Following the feedback, participants completed the puzzle-based measure of motivation, either difficult (Sample 1), playful (Sample 2), or neutral (Sample 3). Finally, participants were given an opportunity to comment on the survey, debriefed, and compensated with $3.00.

### Measures

#### Personality questionnaire: Big Five Aspect Scale

To ascertain the Big Five personality traits, participants completed the 100-item Big Five Aspect Scale (BFAS; [41]). The BFAS is a valid and reliable measure that organizes Big Five scores into ten biologically grounded and meaningful personality aspects (2 aspects per trait). For instance, Neuroticism can be scored as the combined tendency for Volatility or Withdrawal, and Conscientiousness as the combined tendency for Industriousness and Orderliness. Scale reliability and score distributions were comparable across survey conditions (see Table 1).

**Table 1:** Reliability and descriptive statistics for the Big Five Aspect Scale.

	Sample 1: Difficult Condition			Sample 2: Playful Condition			Sample 3: Neutral Condition		
	Mean	SD	Alpha	Mean	SD	Alpha	Mean	SD	Alpha
**Openness**	3.88	.52	.84	3.88	.59	.88	3.88	.62	.89
**Extraversion**	3.20	.72	.91	3.12	.76	.92	3.28	.75	.92
**Conscientiousness**	3.61	.64	.89	3.54	.68	.91	3.66	.65	.90
**Agreeableness**	4.01	.56	.89	3.88	.65	.92	4.01	.63	.91
**Neuroticism**	2.68	.87	.95	2.62	.78	.93	2.58	.89	.95

SD represents standard deviation; Alpha represents Cronbach’s Alpha reliability statistic value.

#### Precursor puzzle and feedback

Prior to the motivational assessment puzzle, participants completed a precursor puzzle based on Raven's Progressive Matrices [63]. This puzzle required participants to complete a pattern presented in a 3x3 grid by choosing 1 of 8 pattern constituents, presented in multiple choice format. The puzzle began with an instructional module which guided participants through two simple practice questions. Real performance feedback was provided in this module, which indicated the accuracy of the selected choice and reminded participants of the rules after inaccurate responses. participants could proceed to the next module once two questions were answered correctly. The second module was comprised of 10 additional matrix puzzles organized in order of increasing difficulty.

After the block containing the precursor puzzle and personality survey was completed, participants were confronted with a page asking them to wait while their results were being processed. In order to increase situational believability, the prompt was accompanied by a rotating animation common to loading web pages. After 15 seconds, the page automatically advanced to one of two randomly assigned prompts. These prompts were identical except for the percentile attributed to the participant. In the positive feedback condition, participants were told that they were among the top 5% of participants. In the negative feedback condition, participants were told that they were among the bottom 5% of participants. Thus, the provided performance feedback was both false and normative. The following represents the prompt in its entirety:

*Your performance on the previous tasks indicates that you are among the [top/bottom 5%] of participants. Because you belong to a sample of individuals that are underrepresented in research, we would like you to complete one more task*.

Several dimensions of this prompt were intentional. For instance, because specific feedback has repeatedly been shown to motivate learning (e.g., [1]), our prompt used *nonspecific* performance feedback [64]. The feedback message was intentionally vague in order to deter participants from inferring any specific tactics for the subsequent puzzle task, based on their responses on the previous segments. Moreover, the subsequent task was portrayed as secondary to the actual experiment in order to encourage the participant to consider the subsequent task as outside of the established motivational domain. Yet, the participant was also reminded that their participation was valuable to the research community, in order to justify their continued effort. This detail was important to include considering that MTurkers are prone to perform more poorly if they feel that their participation is not justified [62]. Finally, participants were informed that the assessment was based on preceding *task performance* in order to communicate that the puzzle task (and not the personality survey) was the basis of the feedback.

This precursor puzzle served two purposes. First and foremost, the puzzle provided a basis for performance feedback (PF), and the reaction of participants to this feedback would be assessed using an alternative puzzle task that measured motivation. Secondly, because it was modeled on Raven's Matrices–the best approximation of fluid-g [65]–the precursor puzzle served as a measure of cognitive ability. Accordingly, performance on this task could be used to control for confounding effects of cognitive ability on the puzzle-based measure of motivation.

#### Discovery task: A puzzle-based measure of motivation

After the feedback prompt, participants were presented instructions for a discovery task. In all three samples, this task entailed searching for and clicking on target images among distractors. All three tasks consisted of 50 pages of images, with valid targets representing 36% of all stimuli (See S1 Supplementary Information for examples). We chose to present more distractors than valid targets so that the tasks would entail active searching rather than passive clicking. In all three samples, participants were notified that some images did not contain valid targets; however, they were not notified of the proportion of valid stimuli. The motivational outcome measure was persistence on these tasks, classically operationalized as the voluntary amount of time spent on the task [66]. Although the percentage of correct discoveries could potentially represent the degree of motivation in each study, this procedure was not favored because it remains unclear whether performance on playful vs. difficult tasks is predicated on comparable motivational processes. Conversely, time on task has been shown to be a valid metric of motivation across many studies in social and personality psychology [67].

In Sample 1 (Difficult Condition), participants completed an embedded figures task which consisted of finding a shape outlined on the right side of the screen within a tangled array of lines on the left side of the screen. In some cases, the embedded shape was a different size than presented, thus requiring participants to mentally re-size the target shape. Upon spotting the shape, participants were instructed to trace the shape within the array by clicking on the apparent corners. Before commencing the task, participants were shown an instructional video demonstrating the procedure. Two task parameters were included in order to facilitate frustration. Firstly, both the target shape and the search area were complexly designed, thereby requiring close inspection and frequent comparison–in contrast to existing published embedded figures tasks. Secondly, only 18 of the 50 images (36%) actually contained a hidden shape. Thus, on 64% of the task, participants were at risk of investing a great amount of search effort without a payoff.

In Sample 2 (Playful Condition), participants worked on a variation of the classic childhood game of "spot-the-difference". Participants were shown two identical or semi-identical cartoon images side by side and asked to click on the objects in the left image that differed from the objects in the right image. The images were shown over 50 pages and only 18 images (36%) contained valid targets. The position of the valid images within the set of images was identical to the distribution of targets in Sample 1 (Difficult Condition). Although we expected the task could be somewhat tedious given the length, and the low number of valid stimuli, we nevertheless expected it to be perceived as playful for two reasons. Firstly, the task represented a childhood game that many of the participants would have played, as it is a common game in the United States. Secondly, the stimuli depicted cheerful cartoon scenarios that were less complex than the stimuli from Sample 1. Thus, the task for Sample 2 was taken to represent a playful puzzle.

In Sample 3 (Neutral Condition), participants completed a variation of the d2 task of attention [68]. This task entailed identifying particular symbols among a set of similar distractor symbols. Specifically, participants were instructed to click on symbols that consisted of the letter b surrounded by two dots at some position while ignoring b's surrounded by a different number of dots, or p's surrounded by any number of dots. The task consisted of 1250 symbols spread over 50 pages, with 450 valid targets (36%) randomly spaced throughout the task. This task was not intended to be particularly frustrating, as the search was not all-or-nothing, as was the case for the task for Sample 1 (Difficult Condition). That is, participants who spent time looking for the targets would eventually find some, if not all, of the targets, thereby justifying their effort. Conversely, this task was not intended to be particularly playful, as the search stimuli were uninteresting and repetitive, unlike the stimuli in Sample 2 (Playful Condition). As such, the task for Sample 3 was taken to represent a neutral puzzle.

In order to verify whether the expected task enjoyment for each sample was actualized, at the end of each survey participants were free to comment on their experience with the task. Comments were optional in order to avoid demand characteristics. The comments were manually coded by a trained research assistant according to sentiment in order to determine the valence associated with each task. The validity of the coding was verified by researchers. The comments were classified as belonging to one of three categories: *irritated–*based on statements of unfairness, frustration, inconvenience, and final relief from tedium–*pleased*–based on statements of gratitude, enjoyment, and amusement–and *neutral*–based on technical statements and general comments. Tasks associated predominantly with irritated or pleased sentiments were taken to represent frustrating or playful tasks, respectively. Surveys with a balance of irritated and pleased sentiments were interpreted as employing neutral tasks.

### Participants

Three convenience samples were iteratively and independently collected through Mechanical Turk. Each sample had a unique stopping rule to account for differing anticipated rates of attrition. The highest stopping rule (*N* = 650) was set for the difficult task survey, followed by the neutral task survey (*N* = 500), and the playful task survey (*N* = 300). Attrition rates for all three surveys were within normal bounds (see [69]), with 33%, 28%, and 15% of participants dropping out of the difficult, playful, and neutral surveys respectively. The sample sizes were further reduced before analyses by excluding participants who asked to withdraw their data, exhibited duplicate IP addresses, failed to pass negligent response checks, or skipped the puzzle task at the end of the survey– in total 14%, 10%, and 8% for the difficult, playful and neutral tasks respectively. Thus, the final total sample size subject to analysis was comprised of 916 participants– 342 in the difficult task condition (58.5% female; *M*_*age*_ = 37, *SD* = 12), 175 in the playful task condition (52.6% female; *M*_*age*_ = 37, *SD* = 11), and 399 in the neutral task condition (61.7% female; *M*_*age*_ = 39, *SD* = 13).

The demographics varied little among the three samples. All participants were resided in the United States, with the majority identifying as Caucasian (81.3%). The total sample included participants who identified as African American (6.2%), Hispanic (5.1%), Asian (4.4%), Native American (1.1%), Pacific Islander (0.2%), and other (1.6%). 62.3% of participants had graduated from college, and 10.9% had a high-school degree alone. 25.9% of participants were students at the time of the survey (8.4% part-time students). The average reported untaxed income for this sample was $40,000 (*SD* = $18,000).

## Results

### Task difficulty perception

Although comments were optional, a substantial proportion of participants who completed each study also provided comments. The coded sentiment of each surveys comments was compared to verify the relative perception of task difficulty. Of the total 120 participants who commented at the end of the difficult task survey, 22% were pleased, 27% were neutral, and 51% were irritated. Given the predominance of irritated comments, this task was confirmed to be particularly frustrating. Of the total 140 participants who commented at the end of the neutral task survey, 33% were pleased, 32% were neutral, and 35% were irritated. Given the relative balance of comment types, this task was confirmed to be neutral (i.e. neither particularly irritating nor satisfying).

Of the total 51 participants who commented at the end of the playful task condition, 49% were pleased, 39% were neutral, and 12% were irritated. Although these proportions are based on a relatively small number of comments, this pattern replicates the proportions found in an unreported sample of 355 participants (57.2% female, *M*_*age*_ = 38 years, *SD* = 12), who completed the exact same task, but without feedback. Of the total 145 participants of this alternative sample who commented at the end of the task, 48% were pleased, 40% were neutral, and 12% were irritated. Taken together, the spot-the-difference task was interpreted to be more playful than frustrating.

### Descriptive statistics

In order to minimize the influence of pauses on persistence scores, time spent on each trial of the puzzle task was compared to time spent on adjacent trials. For instance, time on trial B_i_ was compared to the average time on trial A_i_ and C_i_. If a participant spent five minutes more on trial B_i_ than the average of trial A_i_ and C_i_, trial B_i_ was flagged as a potential pause. On the difficult task, 61 potential pauses were identified out of 17,150 cases; on the playful task, 12 potential pauses were identified out of 8,750 cases. The values of potential pauses were replaced with interpolated values based on the mean of the two adjacent cells, i.e., B_i_ = ((A_i_ + C_i_)/2).

The total time on task for all three survey conditions followed a positively skewed gamma distribution. Thus, in order to identify outliers, and conduct subsequent Gaussian statistics, the total time on puzzle tasks was log-transformed. Box plots identified five outliers in the difficult task condition and two outliers in the playful task condition. With these outliers removed, task time was confirmed to be normally distributed by the Kolmogorov-Smirnov test of normality for both the difficult task condition (*K* = .05, *p* = .08), and the playful task condition (*K* = .05, *p* = .20).

On the neutral task, trial-specific timing information was not collected, thus pauses were inferred from total time outliers. Participants who likely paused were identified by log- and Z-score transforming total time, and considering scores greater than three standard deviations above the mean. Three participants satisfied these criteria and thus were excluded from the final time-based analyses. On average, participants spent 34 minutes completing the difficult task (*SD* = 20 minutes), 25 minutes completing the playful task (*SD* = 13 minutes), and 26 minutes completing the neutral task (*SD* = 11 minutes).

### Feedback manipulation

Positive feedback tends to have a stronger motivational effect than negative feedback. This effect is known to decay, and is strongest immediately after the feedback is received [11]. Accordingly, we conducted three independent samples t-tests to assess whether our feedback manipulation was effective for each of the three surveys. The test variable in these models was the degree of motivation demonstrated on the first five trials of the puzzle task after feedback. The motivational variable represented cumulative time on task for the first five trials of the playful and difficult task surveys. Because we lacked trial specific time data for the neutral task, this motivational variable represented the percentage of correct solutions on the first five trials.

In order to test whether feedback valance affected motivation across tasks, the motivational variables from each study were Z-transformed and combined into a single column.

Across studies, participants who received negative feedback were significantly less motivated (*M* = –.10, *SD* = 1.07, *n* = 438) than those who received positive feedback (*M* = .10, *SD* = .92, *n* = 470); *r* = –.10, *t*(906) = –3.00, *p* = .003. For the condition specific t-test, raw time values were used because the subsequent means are more easily interpretable, with mean values representing average time in minutes. On the playful puzzle task, participants who received negative feedback spent less time on task (*M* = 24.9, *SD* = 11.4, *n* = 85), than participants in the positive feedback condition (*M* = 28.0, *SD* = 13.4, *n* = 89), however this effect was not significant at *p <* .05; *r* = –.12, *t*(172) = –1.64, *p* = .10. On the difficult puzzle task, participants who received negative feedback spent less time on task (*M* = 68.4, *SD* = 48.9, *n* = 161), than participants who received positive feedback (*M* = 76.9, *SD* = 54.9, *n* = 176), though not significantly; *r* = .08, *t*(335) = –1.51, *p* = .13. On the neutral task, participants in the negative feedback condition correctly identified fewer targets (*M* = 97.2, *SD* = 7.4, *n* = 192) than participants in the positive feedback condition (*M* = 98.4, *SD* = 4.2, *n* = 205); *r* = .10 *t*(395) = –2.03, *p* = .04.

Thus, a consistent trend demonstrated across the three surveys suggested that the feedback manipulation was effective, with positive feedback being more motivating than negative feedback. In line with existing research, this pattern was most prominent when free from the confounding effects of task difficulty vs. playfulness, in the neutral task condition.

### Task difficulty x feedback condition

To test whether Big Five scores moderated the motivational effects of *task difficulty x feedback*, a three-way ANOVA was conducted. The dependent variable in this model was the combined time on the playful and difficult tasks (z-transformed). Task difficulty (–1 = difficult condition, 1 = playful condition) and feedback (–1 = negative PF condition) were entered as fixed factors, and the Big Five traits were entered as covariates. Accordingly, the model included five 3-way interaction terms representing *personality x difficulty x feedback*, for each trait (e.g. *Conscientiousness x difficulty x feedback*).

In this model, two main effects and three interactions emerged as significant. More time was spent by participants in the difficult task condition [*F*(1, 487) = 11.82, *p* = .001] and by participants who were more agreeable [*F*(1, 487) = 7.81, *p* = .005]. The three significant interactions were *Conscientiousness x feedback x difficulty* [*F*(3, 487) = 4.08, *p* = .007], *Neuroticism x feedback x difficulty* [*F*(3, 487) = 5.88, *p* = .001], and *Agreeableness x feedback x difficulty* [*F*(3, 487) = 4.21, *p* = .006]. The significance of these three interactions was well below the Bonferroni corrected alpha value for five hypothesis tests (α = 0.5/5 = .01), suggesting that these results were likely not due to Type I error.

To unpack the three-way interactions, two two-way ANOVAs were conducted, one for each feedback condition. In each model, time on task was predicted by difficulty condition, each Big Five trait, and five interactions (*trait x difficulty*). In the negative feedback condition, two interactions emerged as significant: *Conscientiousness x difficulty* [*F*(1, 233) = 11.29, *p* = .001], and *Neuroticism x difficulty* [*F*(1,233) = 14.82, *p <* .001]. In the positive feedback condition, one interaction emerged as significant: *Agreeableness x difficulty* [*F*(1, 253) = 8.62, *p* = .004].

Traditionally, significant two-way interactions are interpreted by conducting two one-way ANOVAs (termed simple effects ANOVAs). For instance, we would test whether time on task is significantly different for high vs. low conscientious people on the difficult task (ANOVA 1), and whether time on task is significantly different for high vs. low conscientious people on the playful task (ANOVA 2). This procedure entails that the independent variable is categorical, however, whereas our personality scores were approximately continuous. Thus, instead of recoding personality into binary categories–which would result in a loss of statistical power–we opted to interpret the significant interactions of the 2-way ANOVAs by comparing regression coefficients. This procedure had the added benefit of providing clear effect sizes for each condition.

To unpack the interactions uncovered in the negative feedback condition, two regressions were conducted predicting time on the difficult task (model 1) and the playful task (model 2). The independent variables in each model were the Big Five personality traits. The significance of differences between coefficients was tested using the following formula: *Z* = (B1 –B2) / √(seB1^2 + seB2^2). Accordingly, conscientious people were found to be significantly more motivated by negative feedback on the playful task (*β* = .45) than on the difficult task (*β* = –.15), *Z = –* 3.45, *p* = .0002. Likewise, neurotic people were found to be significantly more motivated by negative feedback on the playful task (*β* = .32) than on the difficult task (*β* = –.32), *Z* = –3.95, *p* = .00004. Note, as the signs of the beta coefficients suggest, the significant difference between groups was not merely a matter of magnitude; conscientious and neurotic participants were demotivated by negative feedback on the difficult task, and motivated by negative feedback on the playful task.

To elucidate the interactions uncovered in the positive feedback condition, two regressions were conducted predicting time on the difficult task (model 1) and the playful task (model 2). Again, the independent variables in each model were the Big Five traits. Comparison of regressions revealed that agreeable people were significantly more motivated by positive feedback on the playful task (*β* = .34) than on the difficult task (*β* = –.10), *Z* = –2.93, *p* = .002. Although the beta coefficient associated with the difficult task condition was negative, it likely does not represent demotivation because the magnitude was small and it was not a significant contributor to the regression (*p* = .30).

To test for possible mediation effects, task performance was added to the four regressions along with three mediators common to psychological research: fluid-g, age, and gender [70]. Including these variables in the regressions did not change the pattern or significance of the aforementioned results, suggesting that the personality effects were not mediated by common covariates.

To test whether personality interacts with feedback on tasks that are neither difficult nor playful, a two-way ANOVA was conducted with time on the neutral task as the dependent variable. This model was comprised of a total of 11 independent variables: Feedback condition (–1 = negative condition), the Big Five personality traits, and five interaction terms (*feedback x trait*). As in the playful and difficult task conditions, a main effect of Agreeableness emerged with agreeable people spending significantly more time regardless of feedback condition [*F*(1, 360) = 7.40, *p* = .007]. This model contained no significant, or near significant, interaction terms. This finding suggests that interactions between feedback and traits are dependent on the salience of task difficulty.

## Discussion

Across three samples, we found support for our general hypothesis that Conscientiousness and Neuroticism interact with task difficulty to moderate the motivational effects of PF. Specifically, we found that negative feedback produced opposing effects on difficult and playful tasks for conscientious and neurotic people alone. On the difficult task, negative feedback had a demotivating effect for more conscientious and neurotic people. Conversely, on the playful task, negative feedback had a motivating effect for conscientious and neurotic people. When task difficulty was not salient (i.e. in the neutral task condition), negative feedback did not interact with any personality traits.

Given that our hypothesis was informed by Goal Setting Theory and SDT, our results may be understood according to these frameworks. Goal Setting Theorists posit that negative PF provokes a decision-making process that can result in task abandonment or task commitment [21]. While negative feedback inherently communicates that effort must be increased, the probability that effort will actually be increased is dependent on feelings of competence–both theoretically, according to SDT [14], and practically speaking [22]–with greater feelings of competence facilitating greater effort. Difficult tasks undermine feelings of competence, thus negative feedback on difficult tasks tends to have a demotivating effect [17, 18]. Alternatively, playful tasks foster feelings of competence, thus negative feedback on playful tasks tends to have a motivating effect [19, 20]. We suspect that this competence-moderated pattern was pronounced for conscientious and neurotic participants herein because they are inherently more sensitive to competence-related threats [31, 46]. Nevertheless, in order to confirm this explanation, additional research measuring feelings of competence (pre- and post-feedback) is required.

Although we opted to measure the Big Five personality traits, our results suggest that a narrower personality dimension may provide the most parsimonious description of the motivational effects discussed herein: namely, trait perfectionism. Both Conscientiousness and Neuroticism consistently and highly correlate with perfectionism [71], and may play a causal role in the development of perfectionism [72]. Moreover, perfectionism is characterized by a desire to attain high standards and a subsequent sensitivity to competence-related threats [73]. Thus, trait perfectionism may underlie our finding of Conscientiousness and Neuroticism as the sole moderators of *PF x task difficulty*.

In line with this explanation, perfectionists demonstrate an increased sensitivity to negative feedback [74], and feel more negative affect following negative feedback [75]. Moreover, these effects appear to be exacerbated by task difficulty, as perfectionists experience more negative affect after negative feedback on difficult vs. easy tasks [75]. Whether the emotional effects of PF translate into motivational effects for perfectionists remains to be tested.

Outside of the scope of our hypotheses, we found Agreeableness to moderate the motivational effects of positive feedback. Specifically, we found positive feedback to significantly motivate agreeable people working on playful tasks, but not difficult tasks.

Partiality towards positive feedback has been demonstrated for individuals who score highly on Agreeableness-like traits [56], likely because agreeable people tend to favor signals of reward over signals of punishment [76, 77]. This partiality towards positive feedback, combined with a pronounced inclination towards play [78], may have produced the interactions observed with Agreeableness.

Agreeableness also emerged as the only trait to have a main effect on motivation, independent of task condition, or feedback valence. Specifically, agreeable people generally invested more time on all tasks. Agreeable people, in particular, are generally more willing to invest effort for others [79]. This is clearly demonstrated by the fact that agreeable people are more likely to volunteer for occupational tasks in which others are paid workers [80]. This tendency for compliance/helpfulness may have been leveraged by our feedback prompt, which implied that continued participation was important to the scientific community. Indeed, compliance has been found to increase when it is believed to aid the compliance seeker [81].

Collectively, the results from our three studies suggest that there is consistency in the influence of personality traits on the motivational effects of *PF x task difficulty*. However, the certainty of our results is obscured by three classes of limitations pertaining to the feedback, design, and sample employed herein.

Firstly, generalizations from our findings concerning PF are limited, as we considered only one type of PF: false normative non-specific PF. Though theoretically unexpected, our effects may be eliminated or at least reduced when other types of feedback are used, such as true normative PF, false absolute PF, true absolute PF, group-level PF, or multi-source PF. Additional research is required to determine whether Conscientiousness and Neuroticism moderate the motivational effects of *PF x task difficulty* under these alternative feedback conditions.

Similarly, additional research is needed to test whether the feedback effects observed herein emerge following less extreme normative feedback (e.g. "you scored in the top 25%"). We opted to use extreme normative margins at first pass in order to elicit a definite response to the feedback (i.e. to promote or challenge competence). The realization of this goal was evidenced in the consistent motivational differences uncovered in the positive and negative feedback conditions, as well as the condition-consistent themes of participants comments–for instance, in the negative PF condition a participant provided the following remark "Telling me I performed in the bottom 5% of the participants de-motivated me and frustrated me”. Nevertheless, these extreme margins may have influenced some participants to discount the validity of the feedback, or interpret the prompt in unintended ways. For instance, it is possible that participants with extreme personality scores could have interpreted the feedback as confirmation of their extreme scores–e.g. “top 5%” could be interpreted as being in the “top 5%” of neurotic participants–thereby confounding our results. Although we believe this pattern is unlikely because the type of personality scale used herein is relatively low in transparency [82], additional research with different performance margins can rule this out.

In addition to modifying the content of feedback, future research should consider alternative prompt contexts. For instance, the false pretense regarding contribution to the scientific community may have influenced motivational decision-making over and above the feedback valence. This pretense was included to justify additional participation without additional financial compensation, though it may have caused unintended outcomes (such as the main effect of Agreeableness across tasks).

A second class of limitations stems from general design flaws common to the three studies. Firstly, our omission of affect-related measures limits our understanding of participants reactions to feedback. For instance, we are currently unable to determine whether negative feedback on the difficult task resulted in frustration or some other emotion that could reduce motivation (e.g. shame). Although the precise affective response of participants was not pertinent to our hypotheses, future replications that include affect-related measures can shed light on the emotional processes that remain implicit herein.

Similarly, our conclusions regarding the causal mechanism underlying the motivational effects herein remain untested. Although we posit that competence-related threat sensitivity singles Conscientiousness and Neuroticism out as PF moderators, perceived threat was not tested. To establish competence-related threat sensitivity as a pivotal cog in this motivational system, additional research employing self-reported threat sensitivity, or physiological responses to competence-related threat, is warranted.

Finally, our conclusions are obscured by the sampling method that was employed. Specifically, using an online sample for our research introduced two sources of error variance: attrition and decreased supervision. Although we took measures to decrease the influence of attrition on our analyses–such as placing the experimental manipulation late in the survey [69]–attrition rates are nevertheless higher in online samples. Likewise, although measures were taken to decrease the influence of pausing on timing data, laboratory conditions enable a more thorough supervision of participants. Such supervision is particularly important when utilizing time as a critical dependent variable. Accordingly, a replication employing alternative samples is warranted.

## Conclusions

Building on previous findings which demonstrate that personality and task demands individually obstruct the reliability of PF [1], we herein demonstrate that the reliability of PF is further obscured by interactions between personality and task demands. Accordingly, our findings suggest that the reliability of PF may be improved by altering feedback to accommodate task demands and personality. Specifically, our findings suggest that negative feedback can be used to facilitate motivation for conscientious and neurotic people when tasks are playful, though this same feedback can undermine motivation for conscientious and neurotic people when tasks are frustrating.

Although preliminary, our results may be valuable to psychometricians in educational, organizational, and research settings interested in improving the effectiveness of performance feedback. Our results suggest that normative negative PF is beneficial on playful tasks, yet should be used sparingly on difficult tasks, particularly when applied to neurotic and conscientious populations. Accordingly, future researchers may build on our findings to develop and test personalized feedback interventions that can increase the efficacy and reliability of PF.

Moreover, our findings add to the growing body of literature demonstrating the importance of individual differences and situational variables in motivational processes, ultimately lending support to goal-setting theory [83]. More peripherally, our results shed light on the interrelationships between Big Five traits. Specifically, our finding that Neuroticism and Conscientiousness predict the same motivational outcomes to a similar degree suggests that there are important commonalities between these traits which are often shown to conflict [84]. Uncovering this common latent variable (which we suspect is related to feelings of competence) may help us to understand the theoretical underpinnings of the Big Five.

By establishing personality as a consistent moderator of the motivational effects of *PF x task difficultly*, our findings may be used to improve the reliability of PF. The greatest application of our research, however, may be achieved by elucidating the role of mindsets in our results. Our finding that conscientious and neurotic people can be coerced to work harder after negative feedback on playful tasks, for instance, may extend beyond inherently playful tasks. Perhaps simply framing a task as "playful", rather than "frustrating", may produce these results. This idea finds support in research demonstrating that task motivation can be manipulated simply by labeling a task as "difficult" or "easy" [18, 23]. Similarly, directing participants to focus on the process rather than the outcome of a task can transform an otherwise difficult task into a playful task [85]. Thus, motivation may be maximized for conscientious and neurotic people by pairing feedback with task framing, rather than matching feedback to task difficulty. Such findings would have positive implications for psychometricians who are required to use PF with difficult tasks (e.g. in organizational or academic settings).

## Supporting information

S1 Fig Difficult puzzle task example. (PDF)

S2 Fig Playful puzzle task example. (PDF)

S3 Fig Neutral puzzle task example. (PDF)
