# Supplementary figures and images for: Improving the effectiveness of performance feedback by considering personality traits and task demands
Source: PLoS One. 2018 May 22;13(5):e0197810. doi: 10.1371/journal.pone.0197810 (PMC5963754; doi:10.1371/journal.pone.0197810)

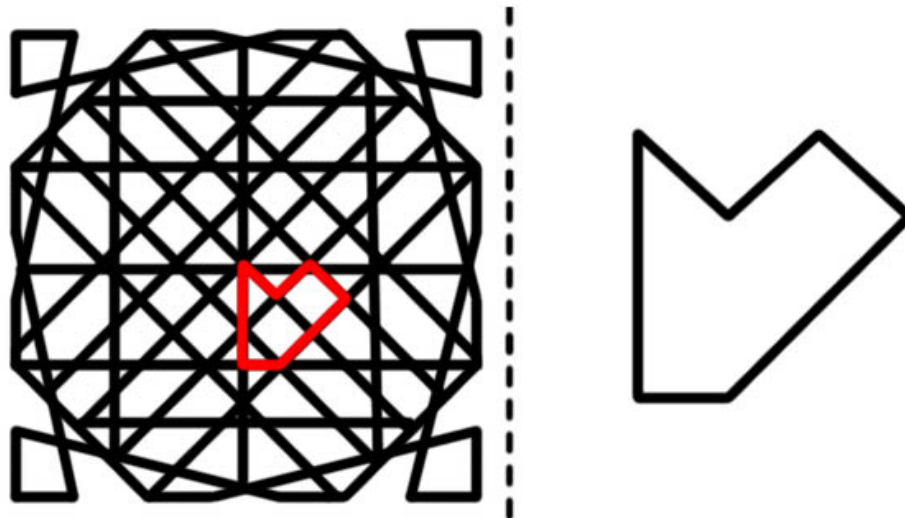

**S1 Fig. Difficult Puzzle Task Example.**

Supplement: S1 Fig — (PDF) [file pone.0197810.s001.pdf]

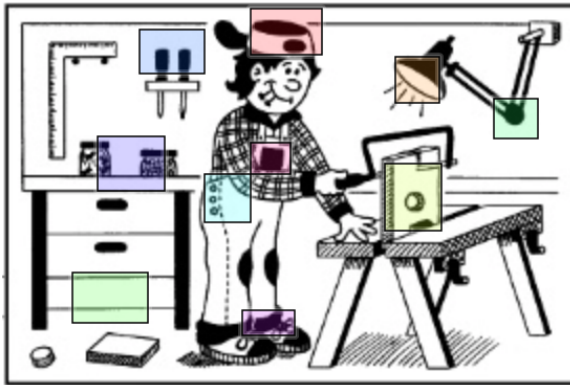

S2 Fig. Playful Puzzle Task Example.

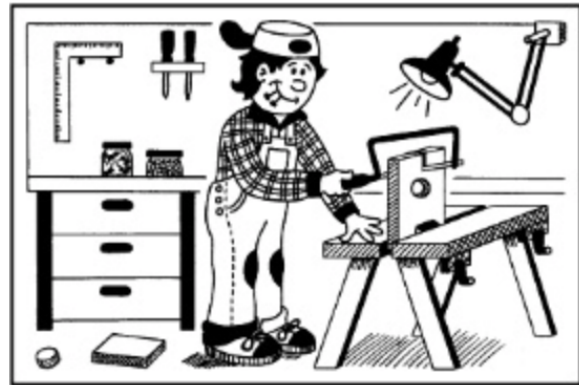

Supplement: S2 Fig — (PDF) [file pone.0197810.s002.pdf]

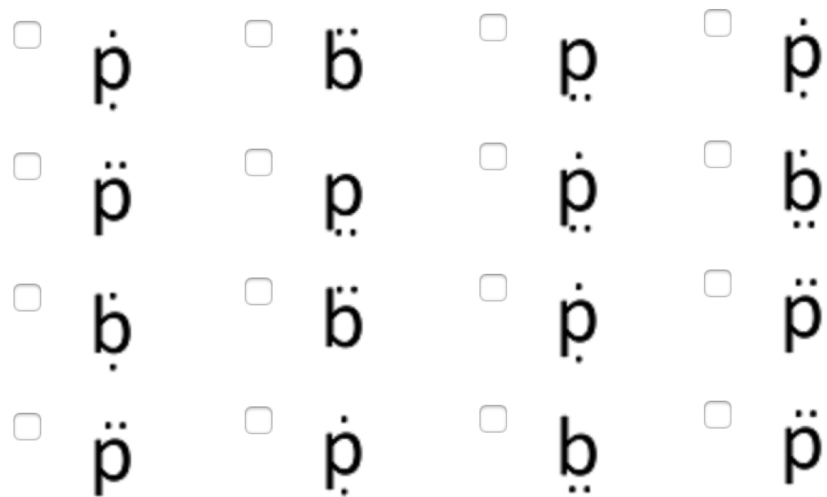

**S3 Fig. Neutral Puzzle Task Example.**

Supplement: S3 Fig — (PDF) [file pone.0197810.s003.pdf]
